# Administration of NaHS Attenuates Footshock-Induced Pathologies and Emotional and Cognitive Dysfunction in Triple Transgenic Alzheimer’s Mice

**DOI:** 10.3389/fnbeh.2015.00312

**Published:** 2015-11-25

**Authors:** Hei-Jen Huang, Shu-Ling Chen, Hsiu Mei Hsieh-Li

**Affiliations:** ^1^Department of Nursing, Mackay Junior College of Medicine, Nursing and ManagementTaipei, Taiwan; ^2^Department of Life Science, National Taiwan Normal UniversityTaipei, Taiwan

**Keywords:** NaHS, anxiety, cognition, footshock stimulus, 3×Tg-AD mice

## Abstract

Alzheimer’s disease (AD) is characterized by progressive cognitive decline and neuropsychiatric symptoms. Increasing evidence indicates that environmental risk factors in young adults may accelerate cognitive loss in AD and that Hydrogen Sulfide (H_2_S) may represent an innovative treatment to slow the progression of AD. Therefore, the aim of this study was to evaluate the effects of NaHS, an H_2_S donor, in a triple transgenic AD mouse model (3×Tg-AD) under footshock with situational reminders (SRs). Inescapable footshock with SRs induced anxiety and cognitive dysfunction as well as a decrease in the levels of plasma H_2_S and GSH and an increase in IL-6 levels in 3×Tg-AD mice. Under footshock with SR stimulus, amyloid deposition, tau protein hyperphosphorylation, and microgliosis were highly increased in the stress-responsive brain structures, including the hippocampus and amygdala, of the AD mice. Oxidative stress, inflammatory response, and β-site APP cleaving enzyme 1 (BACE1) levels were also increased, and the level of inactivated glycogen synthase kinase-3β (GSK3β) (pSer9) was decreased in the hippocampi of AD mice subjected to footshock with SRs. Furthermore, the numbers of cholinergic neurons in the medial septum/diagonal band of Broca (MS/DB) and noradrenergic neurons in the locus coeruleus (LC) were also decreased in the 3×Tg-AD mice under footshock with SRs. These biochemical hallmarks and pathological presentations were all alleviated by the semi-acute administration of NaHS in the AD mice. Together, these findings suggest that footshock with SRs induces the impairment of spatial cognition and emotion, which involve pathological changes in the peripheral and central systems, including the hippocampus, MS/DB, LC, and BLA, and that the administration of NaHS may be a candidate strategy to ameliorate the progression of neurodegeneration.

## Introduction

Alzheimer’s disease (AD) is characterized by neuropsychological symptoms such as anxiety, depression and progressive cognitive deficits along with the deposition of extracellular plaques composed of β-amyloid (Aβ) and intracellular neurofibrillary tangles (NFTs) composed of hyperphosphorylated tau protein. A recent study suggests that psychiatric symptoms such as depression and anxiety increase the severity of cognitive deficits (Ringman et al., 2015). Evidence also indicates that anxiety but not depressive symptoms moderates the effect of Aβ burden on the decline in verbal episodic memory of healthy older adults without dementia (Pietrzak et al., 2014) and in the early stages of AD (Hwang et al., 2004). Frequent or constant stress has also been found to be positively associated with the development of dementia, particularly in the early stages of AD (Johansson et al., 2010; Hebda-Bauer et al., 2013). We previously demonstrated that anxiety induced by inescapable footshock stress associated with situational reminders (SRs) enhances the toxicity of oligomeric Aβ_40_ and also increases the severity of cognitive decline in C57BL/6J mice (Huang et al., 2010). Chronic mild stress increases β-site APP cleaving enzyme 1 (BACE1) levels in the hippocampus of aged mice (O’Connor et al., 2008; Solas et al., 2013). Stress hormones such as glucocorticoids may interfere with the balance between total and phosphorylated tau (Liston and Gan, 2011). Treatment of triple transgenic APP/PS1/tau (3×Tg-AD) mice with dexamethasone, a glucocorticoid receptor agonist, increases brain Aβ levels (Green et al., 2006). In pilot studies, we have demonstrated that male 3×Tg-AD mice exhibit only a mild phenotype at 6 months of age. Therefore, in this study, inescapable footshock with SRs was used to accelerate disease progression in 6-month-old male 3×Tg-AD mice.

Over the last decade, numerous experimental and clinical studies have demonstrated that Aβ plays an important pathogenic role in AD (Lin et al., 2000; Strömberg et al., 2015). Aβ peptides are generated from the sequential proteolytic processing of amyloid precursor protein (APP) by β- and γ-secretases (Strömberg et al., 2015). β-secretase was identified as the transmembrane aspartic protease BACE1 and is responsible for initiating the cleavage of APP (Lin et al., 2000). BACE1 levels and activity are increased in AD (Zhao et al., 2007; Cheng et al., 2014). However, most clinical trials of anti-Aβ therapies have produced little improvement in cognitive function (Castellani and Smith, 2011). Therapeutic strategies that target tau pathology may be more clinically effective than Aβ-directed therapies (Giacobini and Gold, 2013), and very recent studies have suggested that misfolded hyperphosphorylated tau proteins play an important role in AD synaptic dysfunction (Tai et al., 2012; Sokolow et al., 2014). Glycogen synthase kinase-3β (GSK3β) is likely the predominant kinase catalyzing tau protein hyperphosphorylation and subsequent generation of NFTs in AD (Takashima, 2006). However, clinical assessments of GSK3β inhibitors such as lithium or tideglusib have shown no improvement in AD patients (Hampel et al., 2009; Lovestone et al., 2015). Furthermore, long-term treatment with GSK3 inhibitors may be associated with target-related side effects (Tell and Hilgeroth, 2013). These challenges have motivated the development of alternative therapeutic strategies that target the multiple pathogenic cascades of AD (Chen and Zhong, 2013).

Hydrogen sulfide (H_2_S) is an environmental toxin that inhibits cytochrome oxidase (Complex IV) in the mitochondria (Dorman et al., 2002). Three enzymes produce H_2_S in mammalian tissue: cystathionine-b-synthase (CBS), cystathionine-c-lyase (CSE) and 3-mercaptopyruvate sulfur transferase (3MST). CBS is primarily expressed in the central nervous system (Kimura, 2011). H_2_S also has anti-neuroinflammatory (Hu et al., 2007), anti-apoptotic (Yin et al., 2009), antioxidative stress and neuroprotective (Liu and Bian, 2010) effects in mammalian tissue. Endogenous H_2_S levels are severely decreased in the brains of AD patients (Eto et al., 2002), and plasma H_2_S concentrations are negatively correlated with the severity of AD (Liu et al., 2008). Taken together, these findings suggest that H_2_S may protect the brain against footshock with SRs stimulus in 3×Tg-AD mice. Therefore, we examined the neuroprotective effects of NaHS treatment on 3×Tg-AD mice stimulated with footshock with SRs.

## Materials and Methods

### Animals

Pregnant C57BL/6J female mice and 3×Tg-AD (harboring the PS1_M146V_, APP_Swe_ and Tau_P30IL_ transgenes) mice were purchased from the National Breeding Centre for Laboratory Animals and Jackson Laboratory (004807). The phenotype of the male 3×Tg-AD mice (6 months old) was exacerbated by one footshock and four SRs. NaHS or vehicle (saline) was administered before each footshock and SR. The mice were housed in individual ventilation cages at 20–25°C, with free access to food and water and a 12/12-h light/dark cycle (7 AM to 7 PM). This study was conducted in strict accordance with the recommendations of the Guide for the Care and Use of Animals of the National Laboratory Animal Center, Taiwan. The protocol was approved by the Committee on the Ethics of Animal Experiments of the National Taiwan Normal University (Permit Number: 102001).

### Primary Cultures of Mouse Hippocampal Neurons and Drug Treatments

**Figure 1:**
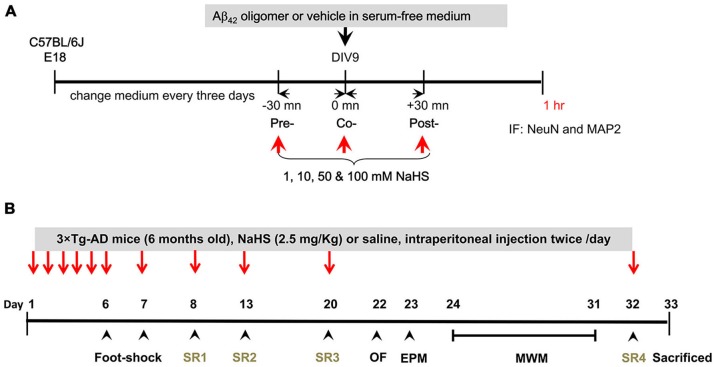
**Schematic diagram of this study. (A)** The experimental timeline for the *in vitro* experiment. Embryonic (E 16–18) hippocampi were isolated for primary culture. At DIV 9, different doses of NaHS (1, 10, 50 or 100 μM) were applied to the hippocampal primary cultures 30 min before (pre-treatment), after (post-treatment), or simultaneously with (co-treatment) the application of oligomeric Aβ_42_. Cells were harvested for IF staining with NeuN and MAP2 antibodies after treatment with oligomeric Aβ_42_ for 1 h. **(B)** The experimental timeline for the *in vivo* treatments and assays. NaHS (arrows) was applied over the first 5 days and each foot shock and SR (arrowheads) on days 8, 13, 20, 32. OF and EPM tests were conducted to measure anxiety levels. The MWM was conducted on days 24–31 to assess the spatial learning and memory of the mice. After SR4, all mice were sacrificed for pathological analyses on day 33.

The method used to prepare primary cell cultures was slightly modified from a previously described protocol (Seibenhener and Wooten, 2012). Briefly, pregnant C57BL/6J mice were sacrificed by cervical dislocation, and the hippocampi were dissected and removed from the brains of embryonic day 16–18 (E16–18) embryos. Tissues were trypsinized (0.05%) for 15 min at 37°C. The cells were plated on 24-well plates (3 × 10^4^ cells per culture well) pre-coated with 100 μg/ml poly-L-lysine and cultured in Neurobasal media (GIBCO, Carlsbad, CA, USA) supplemented with 2% B27 supplement (GIBCO), 0.5 mM glutamine (GIBCO), 25 μM glutamate (Sigma-Aldrich), penicillin/streptomycin (GIBCO, 20 unit/ml), 1 mM HEPES (Sigma-Aldrich), and 1% heat inactivated donor horse serum (GIBCO). The cultures were incubated in a tissue culture incubator at 37°C in 5% CO_2_. On days *in vitro* (DIV) 1, 4 and 7, half of the culture media was replaced with fresh media without horse serum. On DIV 4 and 7, 2 μM cytosine arabinoside (Sigma-Aldrich) was added to reduce the glial cell populations. On DIV 9, the cells were treated with different concentrations (0, 1, 10, 50, or 100 μM) of NaHS (Sigma-Aldrich) and 1 μM oligomeric Aβ_42_ or vehicle at different time points, as shown in the timeline (Figure 1A). Oligomeric Aβ_42_ was prepared as previously described (Kayed et al., 2003), and vehicle was prepared according to the same procedure but without Aβ_42_ powder.

### Immunocytochemistry Analysis

Cells were harvested for immunofluorescence (IF) staining after oligomeric Aβ_42_ administration for 1 h. The cells were first fixed with ice-cold 4% paraformaldehyde (Sigma-Aldrich) for 30 min and washed with phosphate-buffered saline with Triton X-100 (PBST) three times for 10 min each. Non-specific reactivity was blocked by incubation with 10% fetal bovine serum (FBS) for 2 h. The cells were then incubated with NeuN (1:1000; Millipore) and MAP2 (1:1000; Millipore) primary antibodies for 16 h at 4°C, followed by incubation with a secondary antibody for 2 h at 37°C. Finally, neuronal nuclei were counterstained with 4^′^,6-diamino-2-phenylindole (DAPI; Sigma-Aldrich) and immediately analyzed using a High Content Micro-Imaging Acquisition and Screening System (Molecular Devices, Sunnyvale, CA, USA). Both the percentage of neurons and neurite length were analyzed by MetaXpress application software (Molecular Devices).

### Animal Experimental Timeline

The mice received a footshock and SRs as described in a previous study (Huang et al., 2010). As depicted in Figure 1B, the 3 × Tg-AD mice (*n* = 60) were habituated to their home cages and were handled once per day on days 1–5. After adaptation, the mice were placed in the shock chamber (300 × 300 × 350 mm). In the footshock-stimulated group, an inescapable electric footshock was delivered (0.8 mA, 10-s duration, 10-s interval) to the mice after a 120-s acclimation period on days 6 and 7. The non-footshock group did not receive foot shocks. The mice were re-exposed to the same chamber on days 8, 13, 20, and 32, but did not receive foot shock (indicated as SRs 1, 2, 3, and 4). The dose and timing of the NaHS treatment in mice were determined based on results in primary hippocampal neuronal cultures and slightly modified according to a previous study (Zhang et al., 2013). Thus, 2.5 mg/kg (45 μM) was applied *in vivo* via intraperitoneal (ip) injection. To maintain the corresponding concentrations, the treatment was conducted twice a day. The mice received either saline or NaHS (2.5 mg/kg, twice a day) via intraperitoneal injection before footshock and each SR on days 1–8, 13, 20, and 32. The male 3×Tg-AD mice were randomly assigned to four groups (*n* = 15/group): (i) non-footshock/saline; (ii) non-footshock/NaHS; (iii) footshock/saline; and (iv) footshock/NaHS. To evaluate anxiety-like or risk-assessment behaviors, the open-field (OF) test and Elevated plus maze (EPM) were used on days 22–23. On days 24–31, the Morris water maze (MWM) task was conducted, and blood samples were collected to measure the plasma concentration of H_2_S. On day 33, the mice were sacrificed and subjected to pathological characterization using enzyme-linked immunosorbent assay (ELISA), Western blot, and immunohistochemistry analyses.

### Open-Field (OF) Test

The OF test was modified as previously described (Shang et al., 2015). In brief, the OF test was conducted in a white OF box (30 cm × 30 cm × 30 cm) under diffuse lighting. Mice were carefully placed in the center of the box and allowed to explore freely for 5 min in the absence of an observer. After each trial, the box was cleaned, and the animal was returned to the home cage. The arena was partitioned to create a “center” zone (15 cm × 15 cm) and a “corner” zone consisting of the remaining area. A camera mounted on the ceiling above the chamber and connected to an automated video tracking system (EthoVision, Nodulus, Wageningen, Netherlands) was used to capture and quantify images.

### Elevated Plus Maze (EPM)

The EPM consisted of four arms (30 × 5 cm) that were elevated 50 cm above floor level. Two of the arms contained 15-cm-high walls (enclosed arms), while the other two arms remained open (open arms). Each mouse was placed in the middle section facing an open arm and was allowed to explore the maze for a single 5-min session during which the experimenter was out of view. After each trial, the floor was sequentially cleaned with 70% and 30% ethanol. The number of entries, duration spent, and total distances traveled were measured for both types of arms using a video camera and were analyzed with the video tracking system (Nodulus).

### Morris Water Maze (MWM)

The MWM task was conducted as previously described (Huang et al., 2014). Briefly, the system consisted of a circular pool (height 47 cm, diameter 100 cm) filled with opaque water (24 ± 1°C) to a depth of 40 cm. A hidden square platform (10 cm^2^) was submerged 1 cm below the surface of the water in the center of the northeast quadrant as the target quadrant. A video camera was attached to the ceiling to record the behavior of the mice in the pool and was interfaced with EthoVision software (Nodulus).

The experimental procedures included pretraining, training, testing, and probing. During pretraining, the mice were adapted to the pool, and their swimming ability was assessed by allowing them to swim for 60 swithout the platform during each of three 60 s pretraining trials. After each of the three acclimatization trials, the mouse was placed on the hidden platform and allowed to remain on the platform for 20 s. After pretraining, each mouse received training that consisted of 16 trials (four trials per day, lasting a maximum of 60 s each, with an intertrial interval of 20–30 min). The mouse was released into the water from a starting point that randomly varied between trials. When the mouse reached the platform, it was allowed to remain there for 20 s. If the mouse did not find the platform before the 60 s cut-off, it was placed on the platform and allowed to stay there for 20 s. The latency to escape from the water maze (finding the hidden platform) was calculated for each trial. Twenty-four hours after the final training trial, the mice underwent three testing trials to determine the time required to find the hidden platform as a measure of spatial learning acquisition. In the probe trials, which occurred 2 and 48 h after the end of the testing trials, short- and long-term spatial memory were evaluated in the maze with no platform. The probe test was conducted by allowing each mouse to swim freely for 60 s. The time the mice spent in the target quadrant was measured to represent the degree of memory consolidation after learning.

### Immunohistochemistry

The immunohistochemical procedures were performed as previously described (Huang et al., 2012). Briefly, after the behavioral test, the mice (*n* = 3–5 per group) were deeply anesthetized with avertin (0.4 g/kg body weight) and then perfused with 4% paraformaldehyde in 0.1 M PBS. The mouse brains were removed and post-fixed in the same solution at 4°C for 24 h. Then, the brains were dehydrated in a graded series of sucrose solutions until fully permeated. Coronal sections were cut on a cryostat microtome (CMS3050S, Leica Microsystems, Nussloch, Germany) at a thickness of 30 μm. Free-floating sections were incubated with primary antibodies (Table 1) overnight at room temperature, followed by incubation with the appropriate biotinylated secondary antibodies (1:200 dilution, Vector Laboratories, CA, USA) and avidin-biotin-peroxidase complex (Vectastain Elite ABC kit; Vector Laboratories, CA, USA) for 1 h at room temperature. Immunoreactivity was visualized with a diaminobenzidine (DAB) kit (Vector Laboratories, CA, USA). All sections from each group of three to five mice were observed using a light microscope (Leica, Wetzlar, Germany), and images were obtained using the Image-Pro Plus 5.1 image-analysis system (Image Pro Plus Media Cybernetics, Washington, MD, USA). The positive immunohistochemical signal within a specific field was first indicated as the standard signal, and the number of immunopositive cells was then counted using the image-analysis system.

**Table 1:** **List of primary antibodies**.

Antibody	Species	Supplier	WB dilution	IHC dilution
APP	Rabbit	Sigma-Aldrich	1:1000	–
BACE1	Rabbit	Cell Signaling	1:1000	–
5-HT	Rat	Millipore	–	1:200
ChAT	Rabbit	Millipore	–	1:1000
TH	Rabbit	Millipore	–	1:1000
iNOS	Rabbit	Millipore	1:1000	–
MnSOD	Rabbit	Millipore	1:1000	–
GFAP	Mouse	Millipore	–	1:1000
Iba-1	Rabbit	Wako	–	1:1000
CDK5	Mouse	Millipore	1:1000	–
p38	Rabbit	Cell Signaling	1:1000	–
pp38	Rabbit	Cell Signaling	1:1000	–
pS202Tau	Rabbit	AnaSpec	1:1000	–
pS262Tau	Rabbit	Millipore	1:1000	–
pS396Tau	Rabbit	Invitrogen	1:1000	–
pT231Tau	Rabbit	Invitrogen	1:1000	–
GSK3β	Rabbit	Epitomics	1:1000	–
pGSK3β	Rabbit	Epitomics	1:1000	–
CRF	Goat	Santa Cruz	1:1000	–
β-actin	Mouse	Millipore	1:2000	–

*WB, Western blot; IHC, immunohistochemistry*.

### Western Blot Analysis

Isolated hippocampal tissues were homogenized, and the concentration of the isolated proteins was then determined using a bicinchoninic acid (BCA) protein assay kit (Thermo, Rockford, IL, USA) (*n* = 3–5 per group). The homogenates (25 μg of protein) were subsequently separated by SDS-PAGE and transferred to PVDF membranes. The membranes were then blocked in 5% (w/v) skim milk to reduce nonspecific binding and probed with various primary antibodies, as listed in Table 1. The secondary antibodies corresponded to the type of primary antibody and included horseradish peroxidase (HRP)-conjugated anti-mouse or anti-rabbit IgG (1:10,000, Amersham Pharmacia Biotech, Piscataway, NJ, USA). Protein bands were visualized using an enhanced chemiluminescence detection system (Amersham). Protein bands were quantified using an LAS-4000 chemiluminescence detection system (Fujifilm, Tokyo, Japan). The target protein density was normalized to β-actin levels, and protein level changes in the brain tissues of mice treated with footshock are presented relative to the control group. The changes in protein levels refer to control levels to compare experimental conditions.

#### Enzyme-Linked Immunosorbent Assay Analysis

Blood was harvested from a facial vein by lancet after the SRs. The blood was mixed with heparin (20 units/ml) and centrifuged at 2000× g for 20 min at 4°C. The supernatant was collected as a plasma sample to detect the levels of GSH and IL-6 in accordance with the instructions of the glutathione assay kit (Cayman Chemical, Ann Arbor, MI, USA) and IL-6 ELISA kit (R&D Systems, Minneapolis, MN, USA). Mouse Aβ_40_ (KMB3481) and Aβ_42_ (KMB3441) immunoassay kits (Biosource International, Camarillo, CA, USA) were used to determine Aβ_40_ and Aβ_42_ levels in the brain tissues of the mice. ELISAs were performed according to the manufacturer’s protocol as previously described (Huang et al., 2011).

#### Measurement of H_2_S Concentration in Plasma

After the MWM, mouse blood was collected, and plasma H_2_S levels were analyzed as described previously (Zhuo et al., 2009). Plasma (75 μl) was mixed with 250 μl of 1% zinc acetate (Sigma; St. Louis, MO, USA) and 425 μl of water and incubated for 10 min at room temperature. Trichloroacetic acid (250 μl 10% TCA; J.T. Baker, Center Valley, PA, USA) was added to the mixture, which was then centrifuged for 15 min at 14,000× g. The clear supernatant was collected and mixed with equal volumes of 20 mM N-dimethyl-p-phenylenediamine sulfate (Acros Organics, Fair Lawn, NJ, USA) in 7.2 M HCl (Sigma) and 30 mM FeCl_3_ (Sigma) in 1.2 M HCl. After 20 min, the absorbance of the mixture at 670 nm was measured using a microplate reader (Multiskan GO, Thermo). The H_2_S concentration was calculated as previously described (He et al., 2014).

#### Data Analysis

*In vitro* data, data for the OF and MWM tests, and plasma H_2_S levels were compared via two-way analysis of variance (ANOVA) followed by LSD *post hoc* tests for multiple comparisons. The nonparametric EPM data were evaluated using the Kruskal Wallis test. To analyze the pathology measures, ANOVA was performed to compare the parametric data between the three groups, followed by LSD tests for *post hoc* multiple comparisons. SPSS 16.0 software was used to analyze the data. The results are expressed as the mean ± SEM. Differences were considered statistically significant at *p* < 0.05.

## Results

### NaHS Attenuates Oligomeric Aβ_42_-Induced Neurotoxicity in mouse Hippocampal Primary Neuronal Cultures

**Figure 2:**
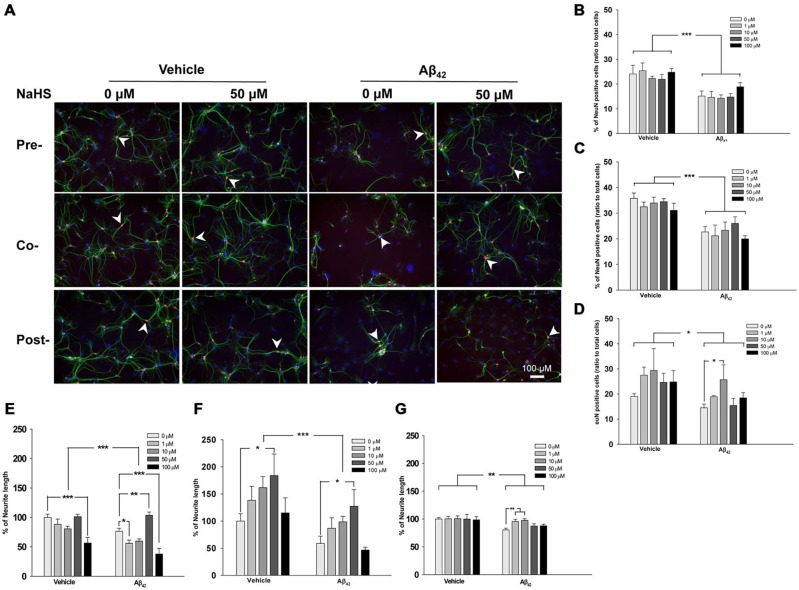
**Timing and doses of the NaHS treatments in mouse hippocampal primary neuronal cultures incubated with oligomeric Aβ_42_. (A)** Representative immunofluorescence images of primary cultures stained for NeuN (red), MAP2 (green) and DAPI (blue). Scale bar: 100 μM. **(B–D)** Quantification of the percentage of mature neurons relative to all cells in oligomeric Aβ_42_-treated primary cultures that also received pre-, co-, and post-treatment with NaHS. **(E–G)** Quantification of neurite length in oligomeric Aβ_42_-treated primary cultures that also received pre-, co-, and post-treatment with NaHS. The arrowheads indicate positive staining (*n* = 3 per group). Data are expressed as the mean ± SEM. *p < 0.05; **p < 0.01; ***p < 0.001.

To evaluate the effect of NaHS on oligomeric Aβ_42_ treated-hippocampal primary neuronal cultures, we applied different doses of NaHS at different times (pre-treatment, post-treatment or co-treatment) relative to the application of oligomeric Aβ_42_. Compared with vehicle treatment, treatment with oligomeric Aβ_42_ significantly decreased the percentage of mature neurons (identified by NeuN staining) when applied pre- (*F*_(1,57)_ = 33.94, *p* < 0.0001; Figures 2A,B), co- (*F*_(1,59)_ = 47.77, *p* < 0.0001; Figures 2A,C), or post- (*F*_(1,57)_ = 5.80, *p* < 0.05; Figures 2A,D) NaHS treatment. Compared with the vehicle treatment, the application of oligomeric Aβ_42_ also significantly decreased neurite length (based on MAP2 staining) when applied pre- (*F*_(1,57)_ = 18.01, *p* < 0.001; Figures 2A,E), co- (*F*_(1,59)_ = 15.18, *p* < 0.001; Figures 2A,F), or post- (*F*_(1,57)_ = 11.98, *p* < 0.01; Figures 2A,G) NaHS treatment. However, pre-, co-, and post-treatment with different doses of NaHS did not affect the percentage of mature neurons compared to saline treatment (*p* > 0.05; Figures 2A–D). In addition, pre-treatment (*F*_(4,57)_ = 19.42, *p* < 0.001; Figures 2A,E) and co-treatment (*F*_(4,59)_ = 4.10, *p* < 0.01; Figures 2A,F) with NaHS significantly increased neurite length compared with saline treatment. There was no significant interaction effect between the oligomeric Aβ_42_ and NaHS treatments on the percentage of mature neurons or neurite length (*p* > 0.05). *Post hoc* multiple comparisons revealed that post-treatment with 10 μM NaHS significantly increased the percentage of mature neurons compared to oligomeric Aβ_42_ treatment (*p* < 0.05; Figure 2D). Neurite length was also significantly increased by 50 μM pre- (*p* < 0.01) and co- (*p* < 0.05) NaHS treatment compared with oligomeric Aβ_42_ treatment. Notably, pre- (*p* < 0.05) and post- (*p* < 0.01) treatment with a low dose (1 or 10 μM) of NaHS significantly increased neurite length compared to saline treatment. However, pre-treatment with a high dose (100 μM) of NaHS significantly decreased neurite length compared to treatment with oligomeric Aβ_42_ (*p* < 0.001). These results suggest that oligomeric Aβ_42_ induces neurotoxicity, whereas NaHS treatment ameliorates oligomeric Aβ_42_-induced neuronal damage.

### NaHS Increases Plasma H_2_S and Ameliorates Anxiety-Like behaviors in Footshock-Stimulated 3×Tg-AD Mice

**Figure 3:**
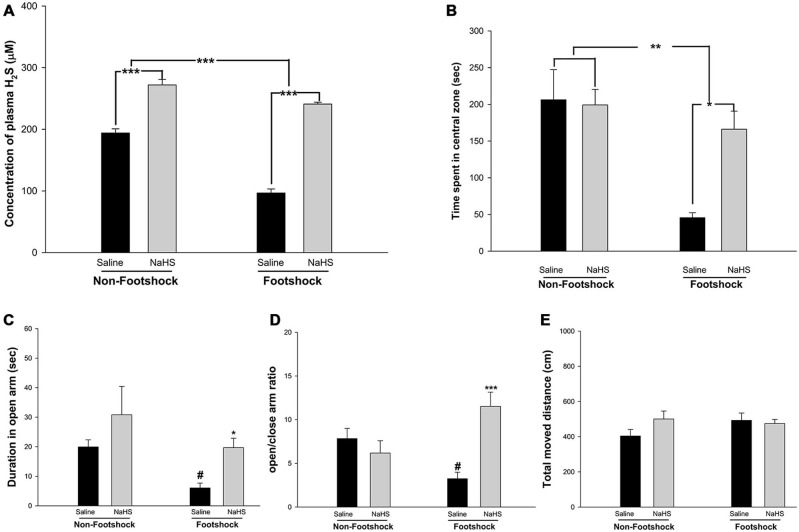
**The effects of NaHS treatment on plasma H_2_S levels and anxiety. (A)** H_2_S concentrations were measured in mouse plasma collected after the MWM. H_2_S levels were reduced in the footshock-stimulated animals, and NaHS treatment recovered the H_2_S levels. **(B)** Time spent in the central zone during the OF test. The animals in the footshock/NaHS group spent more time in the central zone than those in the footshock/saline group. The data are the mean ± SEM of each group (*n* = 15 per group). *p < 0.05; **p < 0.01; ***p < 0.001. **(C,D)** Duration in the open arms and the open/enclosed arm ratio for the four groups. The duration in the open arm and the open/enclosed arm ratio were greater in the footshock/NaHS group than in the footshock/saline group. **(E)** Total distance traveled in the EPM. During the period of the EPM task, the total moved traveled did not differ between any of the groups of mice. Data are mean ± SEM of each group (*n* = 15 per group). ^#^*p* < 0.05, compared to the non-footshock/saline group. *p < 0.05 and ***p < 0.001, compared to the footshock/saline group.

To exacerbate the 3×Tg-AD phenotype, inescapable footshock with SRs was conducted on the mice. Based on the optimal dose (50 μM) identified in the experiments with primary hippocampal neuronal cultures and a previous study (Zhang et al., 2013), we administered 45 μM NaHS (2.5 mg/kg; twice per day) via intraperitoneal injection on days 1–8, 13, 20, and 32. Plasma H_2_S levels were measured after the MWM, and the results demonstrated that footshock significantly reduced plasma H_2_S concentrations (*F*_(1,24)_ = 25.72, *p* < 0.001; Figure 3A). NaHS administration significantly increased plasma H_2_S levels (*F*_(1,24)_ = 77.38, *p* < 0.001; Figure 3A). There was significant interaction between footshock and NaHS (*F*_(1,24)_ = 6.91, *p* < 0.05; Figure 3A). To evaluate the anxiolytic effect of NaHS on the mice, both the OF and EPM tests were conducted. Footshock mice spent significantly less time in the central zone of the OF arena (*F*_(1,19)_ = 15.75, *p* < 0.01; Figure 3B), and NaHS treatment significantly increased the duration spent in the central zone (*F*_(1,19)_ = 5.41, *p* < 0.05; Figure 3B). There was also significant interaction between footshock and NaHS (*F*_(1,19)_ = 6.85, *p* < 0.05; Figure 3B). In the EPM, the frequency with which the footshock-stimulated mice visited the open arms was significantly reduced (*p* < 0.05; Figures 3C,D), and NaHS treatment ameliorated the effect of anxiety, as indicated by the increased amount of time the NaHS-treated mice spent in the open arm (*p* < 0.05; Figure 3C; *p* < 0.001; Figure 3D). There were no significant differences in the total distance traveled between the four groups (*p* > 0.05; Figure 3E). Therefore, footshock with SRs induced anxiety-like behaviors and was associated with reduced plasma H_2_S levels in the 3×Tg-AD mice. The semi-acute administration of NaHS increased plasma H_2_S levels and had an anti-anxiety effect in the footshock-stimulated 3×Tg-AD mice.

### NaHS Reduces Amyloid Deposition and Tau Protein Phosphorylation in the Amygdala in 3×Tg-AD Mice Under Footshock Stimuli

**Figure 4:**
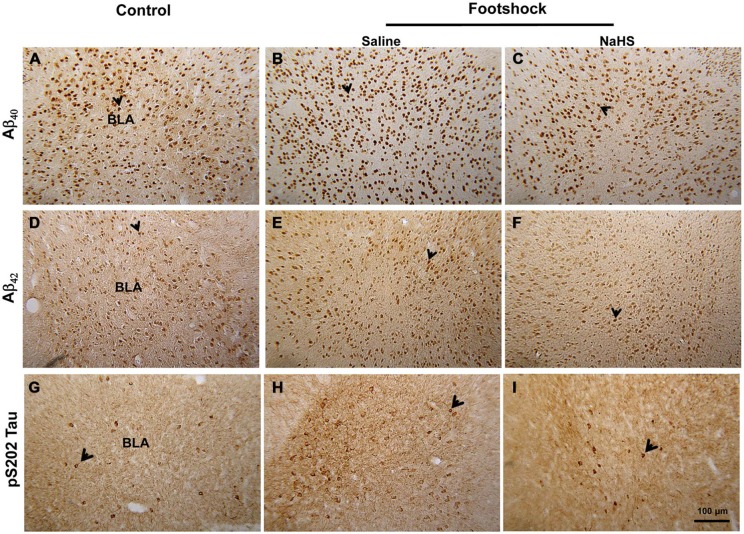
**The effect of NaHS on the level of amyloid deposition and pS202 Tau in footshock-stimulated 3×Tg-AD mice.** Representative immunostaining for Aβ_40_ **(A–C)**. Aβ_42_ **(D–F)** and pS202 Tau **(G–I)** in the BLA. Scale *bar* = 100 μm. The arrowheads indicate positive staining for Aβ_40_, Aβ_42_, and pS202 Tau (*n* = 3–5 per group).

The amygdala mediates anxiety and goal-directed responses (Coutureau et al., 2009; Hubert and Muly, 2014). Immunohistochemical staining revealed that the levels of Aβ_40_ (*F*_(2,15)_ = 16.18, *p* < 0.001; Figure 4 and Table 2), Aβ_42_ (*F*_(2,20)_ = 18.78, *p* < 0.001; Figure 4 and Table 2) and phosphorylated tau protein S202 (pS202 Tau) (*F*_(2,23)_ = 19.45, *p* < 0.001; Figure 4 and Table 2) in the basolateral nucleus of amygdala (BLA) differed significantly between the non-footshock/saline, footshock/saline, and footshock/NaHS groups. In the footshock/saline group, the levels of Aβ_40_ (*p* < 0.001; Figure 4 and Table 2), Aβ_42_ (*p* < 0.001; Figure 4 and Table 2), and pS202 Tau (*p* < 0.001; Figure 4 and Table 2) in the BLA were significantly greater than in the non-footshock/saline (control) group. However, the levels of Aβ_40_ (*p* < 0.001; Figure 4 and Table 2), Aβ_42_ (*p* < 0.001; Figure 2 and Table 2), and pS202 Tau (*p* < 0.001; Figure 4 and Table 2) were significantly lower in the BLA in the footshock/NaHS group than in the footshock/saline group. These results indicate that amyloid deposition and phosphorylated tau protein levels were increased in the footshock-treated mice, whereas semi-acute treatment with NaHS attenuated the footshock-induced pathological changes in the BLA in 3×Tg-AD mice.

**Table 2:** **Immunohistochemistry results for mice receiving different treatments**.

	**Control**	**Footshock/Saline**	**Footshock/NaHS**
Aβ_40_ in CA1	71.86 ± 1.14	118.25 ± 1.12^a^***	72.63 ± 1.75^b^***
Aβ_40_ in BLA	114.00 ± 7.00	161.25 ± 14.28^a^***	103.14 ± 15.38^b^***
Aβ_42_ in CA1	41.71 ± 1.78	82.40 ± 0.94^a^***	47.89 ± 1.26^b^***
Aβ_42_ in BLA	61.71 ± 2.22	163.00 ± 4.15^a^***	82.22 ± 2.66^b^***
PS202 Tau in CA1	14.00 ± 0.70	26.50 ± 0.35^a*^	6.25 ± 0.35^a*, b^***
PS202 Tau in BLA	26.27 ± 0.88	59.20 ± 0.12^a^***	27.88 ± 0.11^b^***
Iba1 in hippocampus	41.75 ± 0.88	58.67 ± 0.98^a**^	29.42 ± 0.88^a**, b ^***
Iba1 in amygdala	32.55 ± 0.53	67.67 ± 0.68^a^***	39.17 ± 0.63^b^***
GFAP in hippocampus	16.83 ± 0.48	43.20 ± 1.02^a^***	19.20 ± 0.83^b^***
ChAT in MS/DB	191.38 ± 4.44	122.75 ± 1.87^a**^	222.00 ± 1.89^b^***
TH in LC	63.80 ± 1.13	49.40 ± 0.64^a*^	70.75 ± 0.86^b**^

Each value represents the mean ± SEM (*n* = 3–5 per group).^a^compared to the control group.^b^compared to the footshock/saline group. CA1, CA1 subregion of the hippocampus; BLA, basolateral nucleus of the amygdala; ChAT, Cholinergic-reactive neurons; MS/DB, medial septum/diagonal band of Broca; TH, noradrenergic-reactive neurons; LC, locus coeruleus. *p < 0.05; **p < 0.01; ***p < 0.001.

### NaHS Attenuates the Impairment of Spatial Learning and Memory in 3×Tg-AD Mice under Footshock Stimuli

**Figure 5:**
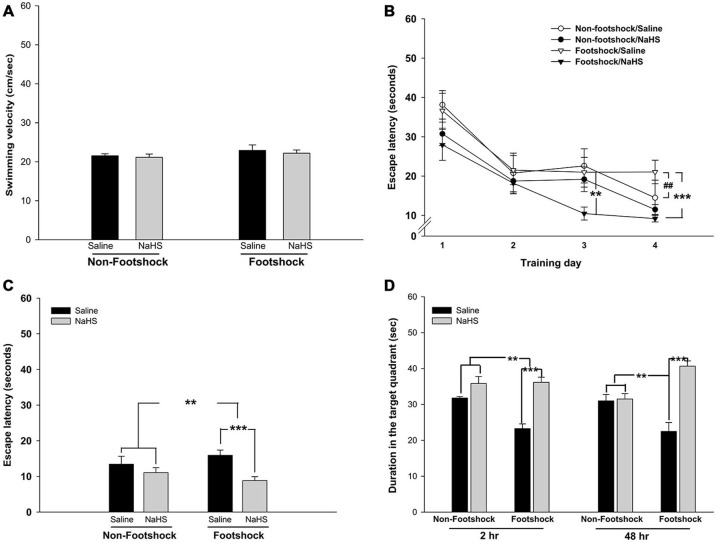
**NaHS treatment attenuated the impairment of cognition in footshock-stimulated 3×Tg-AD mice. (A)** Swimming velocity of the mice in the MWM. The mice in all groups had the same swimming ability. **(B)** Learning trends of the mice over the 4 training days of the MWM. Footshock accelerated the impairment of spatial learning ability and acquisition, and NaHS reduced the impairment in the footshock group. **(C)** The latency of mice to find the platform in the testing trials. The mice under footshock took longer to climb onto the platform than the control mice, and NaHS treatment ameliorated the impairment in the footshock group. **(D)** The results of short- and long-term memory retrieval. A probe trial was conducted 2 and 48 h after the last testing trial to evaluate short- and long-term memory retrieval. Footshock enhanced the impairment of short- and long-term memory retrieval, and NaHS attenuated the deficit. The data are the mean ± SEM of each group (*n* = 15 per group). **p < 0.01; ***p < 0.001. ^##^*p* < 0.01 for comparisons with the non-footshock/saline group.

Of the regions in the brain, the hippocampus is the most vulnerable to stressful conditions (Semmler et al., 2005).To investigate the effects of footshock and/or NaHS treatment on spatial cognition, we employed the MWM task to examine hippocampus-dependent learning and memory. All animals swam normally during the pretraining trials (*p* > 0.05; Figure 5A). During the training phase of the MWM, the footshock/saline group did not exhibit a significant decrease in escape latency onto the platform following training days 1–4 (*p* = 0.07; Figure 5B); however, the escape latency of the footshock/NaHS group was significantly decreased (*F*_(3,39)_ = 11.36, *p* < 0.001; Figure 5B). By contrast, the escape latency onto the platform of the non-footshock group was also significantly decreased with saline (*F*_(3,27)_ = 14.47, *p* < 0.001; Figure 5B) and NaHS (*F*_(3,15)_ = 16.185, *p* < 0.001; Figure 5B) treatment. The escape latency of the footshock/saline group on training day 4 was significantly greater than that of the non-footshock/saline group (*p* < 0.01; Figure 5B). However, the escape latency of the footshock/NaHS group on training days 3 (*p* < 0.01; Figure 3B) and 4 (*p* < 0.001; Figure 5B) was significantly decreased compared to that of the footshock/saline group.

During the test period, the time spent searching for the platform was assessed as an indicator of the acquisition of spatial learning. The spatial learning acquisition was significantly lower in the footshock group than in the non-footshock group (*F*_(1,33)_ = 2.79, *p* < 0.01; Figure 5C). However, compared with saline treatment, NaHS treatment significantly attenuated the deficit in spatial learning acquisition (*F*_(1,33)_ = 3.54, *p* < 0.001; Figure 5C). There was also a significant interaction between footshock and NaHS in spatial learning acquisition (*F*_(1,33)_ = 4.5, *p* < 0.001; Figure 5C). *Post hoc* multiple comparisons revealed that the deficit in spatial learning acquisition was significantly lower in the footshock/NaHS group than in the footshock/saline group (*p* < 0.001; Figure 5C).

Two hours after the end of the testing trials, retrieval of short-term memory was significantly impaired in the footshock group compared to the non-footshock group (*F*_(1,20)_ = 7.43, *p* < 0.05; Figure 5D). The administration of NaHS significantly reduced the deficit in short-term memory retrieval compared with saline treatment (*F*_(1,20)_ = 31.62, *p* < 0.001; Figure 5D). There was also a significant interaction between footshock and NaHS (*F*_(1,20)_ = 8.68, *p* < 0.01; Figure 5D). Forty-eight hours after the end of the testing trials, the retrieval of long-term memory was further assessed. An impairment of long-term memory retrieval was not observed in the footshock group compared to the non-footshock group (*p* = 0.85; Figure 5D). However, NaHS treatment significantly prevented the deficit in long-term memory retrieval compared to saline treatment (*F*_(1,20)_ = 28.66, *p* < 0.001; Figure 5D). In addition, there was a significant interaction between footshock and NaHS (*F*_(1,20)_ = 25.67, *p* < 0.001; Figure 5D). *Post hoc* multiple comparisons revealed that the footshock/saline group exhibited significant impairment of long-term memory retrieval compared with the non-footshock/saline group (*p* < 0.001; Figure 5D). These results indicate that footshock with SRs exacerbated the impairment of spatial learning and memory and that semi-acute treatment with NaHS ameliorated these impairments in the footshock-stimulated 3×Tg-AD mice. However, the semi-acute administration of NaHS had no effect on cognition or emotion in the 3×Tg-AD mice without footshock. Therefore, three groups of mice, non-footshock/saline (control), footshock/saline, and footshock/NaHS, were subjected to further pathological analyses.

### NaHS Strongly Attenuates Amyloidogenesis in the Hippocampus in 3×Tg-AD Mice under Footshock Stimulus

**Figure 6:**
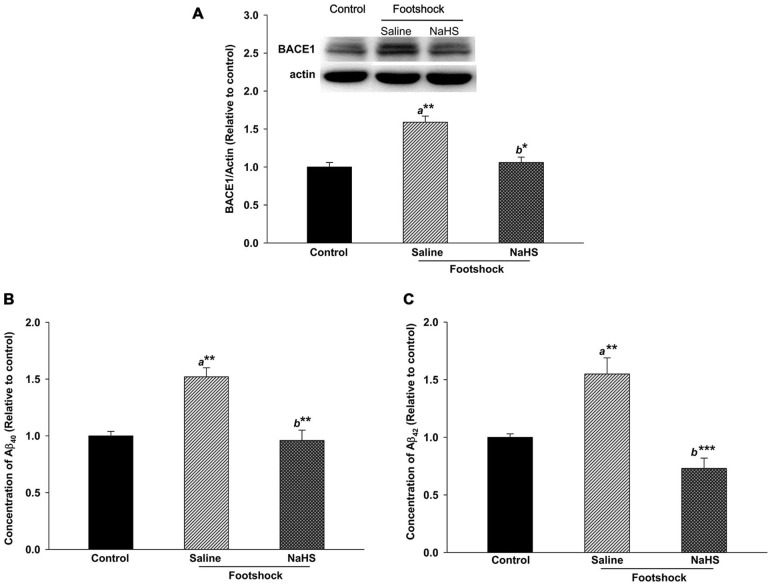
**NaHS treatment decreased the levels of Aβ and BACE1 in the hippocampi of mice under footshock stimulus. (A)** Representative Western blot and densitometry for BACE1 in the mouse hippocampus. β-Actin was used as the internal control. **(B)** Aβ_40_ levels in the mouse hippocampus analyzed by ELISA. **(C)** Aβ_42_ levels in the mouse hippocampus measured by ELISA. The data are the mean ± SEM of each group (*n* = 3–5 per group). *p < 0.05; **p < 0.01; ***p < 0.001. *a*, compared to the control group. *b*, compared to the footshock/saline group.

**Figure 7:**
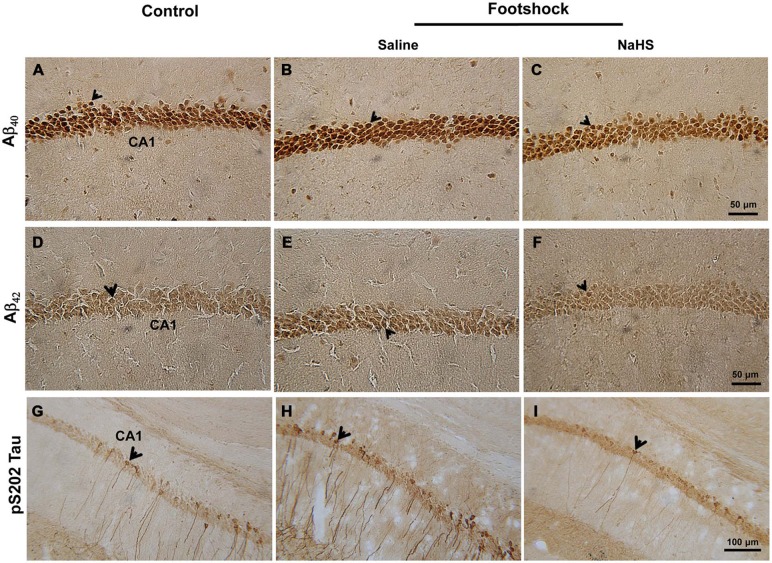
**NaHS decreased the levels of Aβ and phosphorylated tau protein in the hippocampal CA1 subregion of the footshock-stimulated mice. (A–F)** Representative immunostaining for Aβ_40_ and Aβ_42_ in area CA1 of the mouse hippocampus. Scale *bar* = 50 μm. **(G–I)** Representative immunostaining for tau protein phosphorylated at S202 (pS202 Tau) in area CA1 of the hippocampus. Scale *bar* = 100 μm. The arrowheads indicated positive staining for Aβ_40_, Aβ_42_, and pS202 Tau (*n* = 3–5 per group).

The level of Aβ deposition in the hippocampus plays an important role in the loss of cognitive function in AD. Therefore, we examined BACE1 and Aβ levels in the hippocampus in 3×Tg-AD mice. BACE1 expression differed significantly between the three groups (*F*_(2,8)_ = 8.97, *p* < 0.05; Figure 6A). A significantly higher level of BACE1 was observed in the footshock/saline group compared to the control group (*p* < 0.01; Figure 6A). However, the level of BACE1 was significantly decreased in the footshock/NaHS group compared to the footshock/saline group (*p* < 0.05; Figure 6A). Guanidine-soluble Aβ_40_ and Aβ_42_ levels in the hippocampus were measured using a sensitive sandwich ELISA, which revealed significant differences in Aβ_40_ (*F*_(2,8)_ = 44.03, *p* < 0.001; Figure 6B) and Aβ_42_ (*F*_(2,8)_ = 13.72, *p* < 0.01; Figure 6C) levels between the three groups. In addition, the levels of Aβ_40_ and Aβ_42_ were significantly greater in the footshock/saline group than in the control group (*p* < 0.01; Figures 6B,C), and NaHS treatment reduced the levels of Aβ_40_ (*p* < 0.01; Figure 6B) and Aβ_42_ **(***p* < 0.001; Figure 6C) compared to the footshock/saline group. The levels of Aβ_40_ (*F*_(2,18)_ = 16.18, *p* < 0.001; Figure 7 and Table 2) and Aβ_42_ (*F*_(2,20)_ = 13.87, *p* < 0.001; Figure 7 and Table 2) in the CA1 subregion of the hippocampus differed significantly between the three groups. *Post hoc* LSD multiple comparisons indicated that footshock significantly increased the level of Aβ in area CA1 of the hippocampus compared with the Aβ level in the control group (*p* < 0.001; Figure 7 and Table 2), and the level of Aβ in area CA1 of the hippocampus was significantly lower in the footshock/NaHS group than in the footshock/saline group (*p* < 0.001; Figure 7 and Table 2). These results suggest that semi-acute treatment with NaHS attenuated amyloidogenesis in the hippocampus in association with a reduced level of hippocampal BACE1 in the 3×Tg-AD mice under footshock stimulus.

### NaHS Reduces Tau Protein Hyperphosphorylation Associated with Increased pS9-GSK3β in 3×Tg-AD Mice under Footshock Stimulus

**Figure 8:**
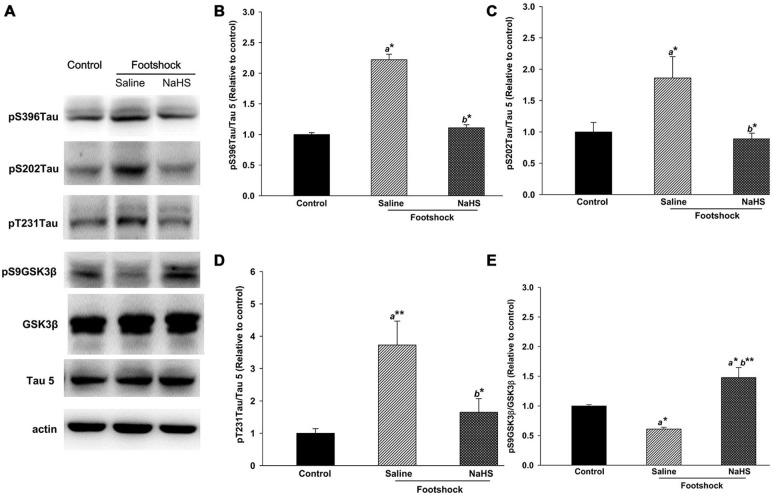
**NaHS decreased the levels of various forms of phosphorylated tau protein and increased the level of pS9GSK3β in the hippocampus of mice under footshock.** Representative Western blots **(A)** and densitometry for phosphorylated tau (Ser396; **B**), (Ser202; **C**), and (Thr231; **D**) as well as the pS9GSK3β/GSK3β ratio **(E).** Tau 5 was used for total tau, with β-actin as the internal control. The quantitative data are the mean ± SEM of each group (*n* = 3–5 per group). *p < 0.05; **p < 0.01. *a*, compared to the control group.* b*, compared to the footshock/saline group.

Immunohistochemical staining demonstrated that, in addition to Aβ levels, the levels of pS202 Tau in area CA1 of the hippocampus differed significantly between the three groups (*F*_(2,14)_ = 14.42, *p* < 0.001; Figure 7 and Table 2). Furthermore, footshock significantly increased the level of pS202 Tau in area CA1 of the hippocampus (*p* < 0.05; Figure 7 and Table 2), whereas NaHS treatment in footshock-stimulated mice significantly reduced pS202 Tau levels (*p* < 0.001; Figure 7 and Table 2). Hippocampal levels of various forms of phosphorylated tau protein were further characterized by Western blot. We observed significant differences between the three groups in the levels of tau protein phosphorylated at S396 (*F*_(2,8)_ = 6.94, *p* < 0.05; Figures 8A,B), S202 (*F*_(2,8)_ = 5.70, *p* < 0.05; Figures 8A,C), and T231 (*F*_(2,8)_ = 9.55, *p* < 0.05; Figures 8A,D). The levels of tau protein phosphorylated at S396 (*p* < 0.05; Figures 8A,B), S202 (*p* < 0.05; Figures 8A,C) and T231 (*p* < 0.01; Figures 8A,D) were significantly increased in the footshock/saline group compared to the control group. However, the levels of tau protein phosphorylated at S396 (*p* < 0.05; Figures 8A,B), S202 (*p* < 0.05; Figures 8A,C), and T231 (*p* < 0.05; Figures 8A,D) were significantly reduced in the footshock/NaHS group compared to the footshock/saline group. Regarding the kinases that phosphorylate tau protein, we observed significant differences between the three groups in the inactive form of GSK3β (pS9-GSK3β) (*F*_(2,8)_ = 18.47, *p* < 0.01; Figures 8A,E) but not CDK5 (data not shown). The pS9-GSK3β/GSK3β ratio was significantly lower in the footshock/saline group compared to the control group (*p* < 0.05; Figures 8A,E) but significantly higher in the footshock/NaHS group compared to the footshock/saline (*p* < 0.01; Figures 8A,E) and control (*p* < 0.05; Figures 8A,E) groups. Therefore, footshock with SRs increased the levels of phosphorylated tau proteins in the hippocampus, but the semi-acute administration of NaHS considerably reduced the tau hyperphosphorylation associated with increasing hippocampal pS9-GSK3β levels in the footshock-stimulated 3×Tg-AD mice.

### NaHS Decreases Inflammatory Responses, Oxidative Stress, and Gliosis in 3×Tg-AD Mice under Footshock Stimulus

**Figure 9:**
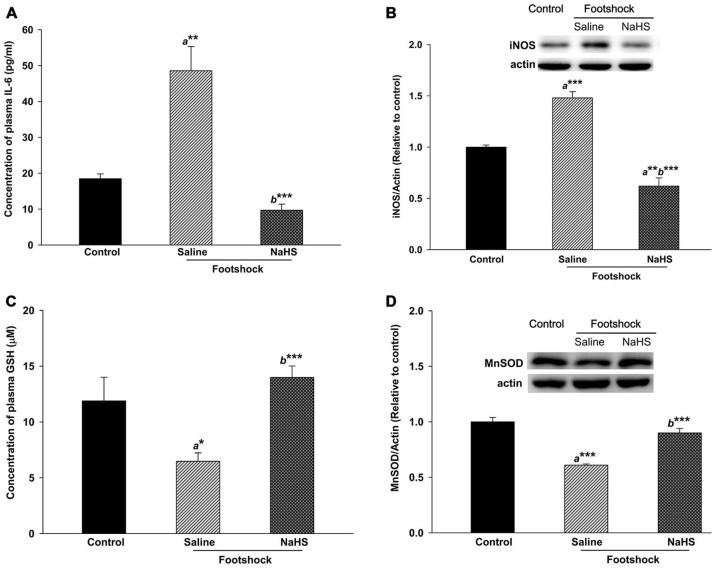
**Antioxidative and anti-inflammatory activity of NaHS in mice under footshock. (A)** Levels of the inflammatory cytokine IL-6 in mouse plasma analyzed by ELISA. **(B)** Representative Western blot and densitometry for iNOS in the mouse hippocampus. Footshock strongly induced IL-6 and iNOS, and NaHS ameliorated the inflammation. **(C)** Concentrations of GSH in mouse plasma measured by ELISA. **(D)** Representative Western blot and densitometry for MnSOD in the hippocampus. Footshock reduced the levels of GSH and MnSOD, and NaHS improved the antioxidant capacity of the mice under footshock. β-Actin was used as an internal control. The data are the mean ± SEM of each group (*n* = 3–5 per group). *p < 0.05; **p < 0.01; ***p < 0.001. *a*, compared to the control group. *b*, compared to the footshock/saline group.

Oxidative stress and inflammation in both the hippocampus and plasma were also evaluated. In the three groups, there were significant differences in the levels of IL-6 in plasma (*F*_(2,10)_ = 22.14, *p* < 0.001; Figure 9A), iNOS in the hippocampus (*F*_(2,8)_ = 56.15, *p* < 0.001; Figure 9B), GSH in plasma (*F*_(2,17)_ = 9.55, *p* < 0.05; Figure 9C), and MnSOD in the hippocampus (*F*_(2,8)_ = 37.31, *p* < 0.001; Figure 9D). Compared with the control group, the footshock/saline group exhibited significant increases in IL-6 in plasma (*p* < 0.01; Figure 9A) and in iNOS in the hippocampus (*p* < 0.001; Figure 9B) and significant decreases in plasma GSH (*p* < 0.05; Figure 9C) and hippocampal MnSOD (*p* < 0.001; Figure 9D). We also detected lower levels of plasma IL-6 (*p* < 0.001; Figure 9A) and hippocampal iNOS (*p* < 0.001; Figure 9B) and higher levels of plasma GSH (*p* < 0.001; Figure 9C) and hippocampal MnSOD (*p* < 0.001; Figure 9D) in the footshock**/**NaHS group compared to the footshock**/**saline group. The level of hippocampal iNOS was lower in the footshock/NaHS group than the control group (*p* < 0.01; Figure 9B). In addition, our results demonstrated that footshock with SRs did not alter inflammatory-related pathways, including COX2, NF-κB, and MAPK, in the hippocampi of 3×Tg-AD mice (data not shown).

**Figure 10:**
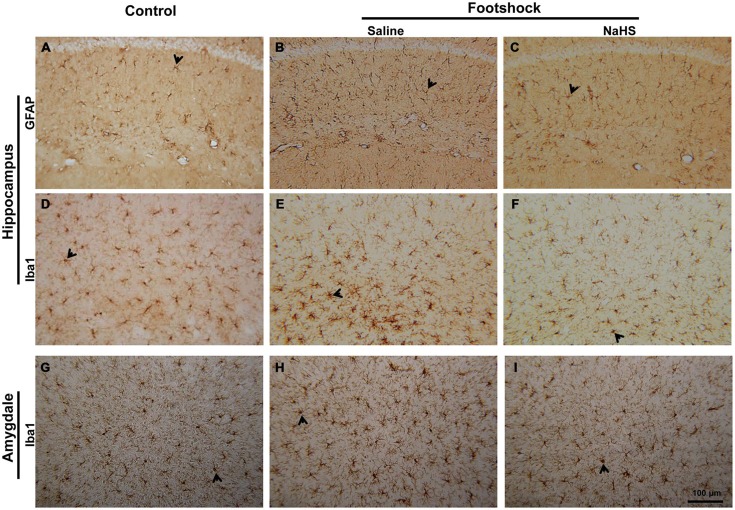
**NaHS reduced neuroinflammation in mice with footshock. (A–C)** Representative immunostaining of activated astrocytes (GFAP) in the CA1 subregion of the mouse hippocampus. **(D–F)** Representative immunostaining of activated microglia (Iba1) in the CA1 subregion of the mouse hippocampus. **(G-I)** Representative immunostaining of activated microglia (Iba1) in the basolateral nucleus of the mouse amygdala (BLA). Scale *bar* = 100 μm. Arrowheads indicated positive staining for GFAP and Iba1 (*n* = 3–5 per group).

We further investigated whether neuroinflammation in the hippocampus and amygdala was affected. The number of reactive astrocytes in the hippocampus differed significantly between the three groups of mice (*F*_(2,15)_ = 18.23, *p* < 0.001; Figure 10 and Table 2). The numbers of reactive microglia in the hippocampus (*F*_(2,25)_ = 21.19, *p* < 0.001; Figure 10 and Table 2) as well as in the BLA (*F*_(2,22)_ = 69.25, *p* < 0.001; Figure 10 and Table 2) also differed significantly between the three groups of mice. The number of reactive astrocytes in the hippocampus of the footshock/saline group was increased compared to the control group (*p* < 0.001; Figure 10 and Table 2). The numbers of reactive microglia in the hippocampus (*p* < 0.01; Figure 10 and Table 2) and BLA (*p* < 0.001; Figure 10 and Table 2) were also increased in the footshock/saline group compared with the control group. However, compared with the numbers in the footshock/saline group, significantly fewer activated astrocytes (*p* < 0.001; Figure 10 and Table 2), microglia in the hippocampus (*p* < 0.001; Figure 10 and Table 2), and microglia in the BLA (*p* < 0.001; Figure 10 and Table 2) were observed in the footshock/NaHS group. We also observed a decreased number of reactive microglia in the hippocampus in the footshock/NaHS group compared to the control group (*p* < 0.01; Figure 10 and Table 2). Therefore, the semi-acute administration of NaHS decreased gliosis in the hippocampus and amygdala and reduced inflammation and oxidative stress in the footshock-stimulated 3×Tg-AD mice.

### NaHS Prevents Cholinergic and Noradrenergic Neuronal Loss in 3×Tg-AD Mice under Footshock Stimulus

**Figure 11:**
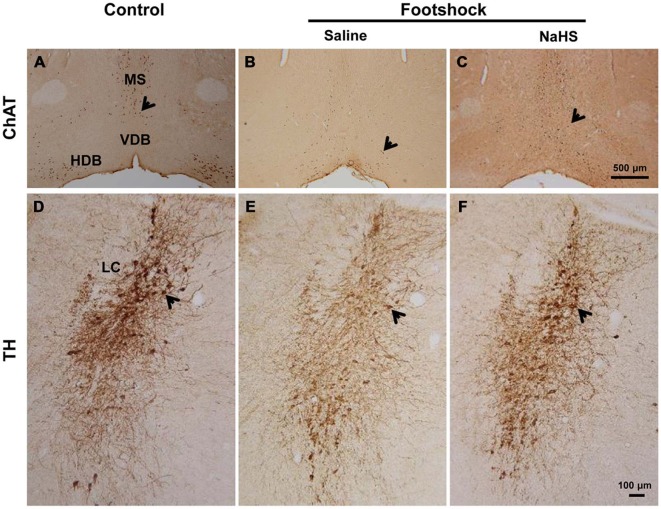
**NaHS prevented cholinergic and noradrenergic neuronal loss in mice under footshock. (A-C)** Representative photomicrographs of cholinergic neurons (ChAT staining) in the medial septum/diagonal band of Broca (MS/DB). Scale bar: 500 μM. **(D-F)** Representative photomicrographs of noradrenergic neurons (TH staining) in the locus coeruleus (LC). Scale *bar* = 100 μm. Positive staining is indicated by arrows. *n* = 3–5 per group.

In the three groups, we observed significant differences in the number of cholinergic neurons in the MS/DB (*F*_(2,15)_ = 8.97, *p* < 0.01; Figure 11 and Table 2**)** and of noradrenergic neurons in the LC (*F*_(2,13)_ = 6.67, *p* < 0.05; Figure 11 and Table 2). Fewer cholinergic (*p* < 0.01; Figure 11 and Table 2) and noradrenergic (*p* < 0.05; Figure 11 and Table 2) neurons were observed in the footshock/saline group compared with the control group. However, compared with the footshock/saline group, cholinergic (*p* < 0.001; Figure 11 and Table 2) and noradrenergic neuronal loss (*p* < 0.01; Figure 11 and Table 2) did not occur in the footshock/NaHS group. However, serotonergic neurons in the raphe nucleus and pyramidal neurons in the hippocampus were not altered in the mice that received footshock or NaHS treatment (data not shown). These results suggest that the semi-acute administration of NaHS prevented cholinergic and noradrenergic neuronal loss in the footshock-stimulated 3×Tg-AD mice.

## Discussion

In this study, we demonstrated that treatment with NaHS attenuated oligomeric Aβ_42_-induced neurotoxicity in hippocampal primary neuronal cultures. *In vivo* studies indicated that footshock with SRs induced the impairment of spatial cognition and emotion, which are involved in peripheral and central systemic deficits including the hippocampus, MS/DB, LC, and amygdala regions, whereas emotional and cognitive dysfunction were attenuated by semi-acute treatment with NaHS through targeting multiple pathogenic cascade in the 3×Tg-AD mice.

First, we observed that pre- and co-treatment with 50 μM of NaHS counteracted the decrease in neurite length caused by oligomeric Aβ_42_-induced neurotoxicity. Pretreatment with a 2.5 mg/kg dose of NaHS can effectively reduce lesion volume induced by traumatic brain injury (Zhang et al., 2013). In addition, increasing the concentration of NaHS did not increase the effect on neurite length. The administration of high doses of NaHS of 10 mg/kg induces systemic inflammation in mice (Bhatia et al., 2006; Zhang et al., 2007). Therefore, 2.5 mg/kg NaHS was used in subsequent animal experiments.

Environmental risk factors due to inescapable footshock with SRs increased anxiety-like behaviors as well as AD pathology such as gliosis, amyloid deposition, and tau hyperphosphorylation in the hippocampus and amygdala of 3×Tg-AD mice. The hippocampus, amygdala, and prefrontal cortex undergo stress-induced structural remodeling, which alters behavioral and physiological responses (McEwen, 2013). In addition, the hippocampus is the most vulnerable region of the brain during stressful conditions (Semmler et al., 2005), and a role for the amygdala in the onset of anxiety behaviors has been demonstrated (Poulos et al., 2009) The amygdala is a component of the limbic system and is involved in emotion and a variety of cognitive functions due to its extensive connections with many brain areas, such as the hippocampus (Yao et al., 2013). Tauopathy begins in the CA1 subregion of hippocampus in 3×Tg-AD mice (Oddo et al., 2003), followed by the progression of tauopathy to other areas such as the amygdala (Yamin, 2009). Therefore, we evaluated the performance of these mice on the OF and EPM as well as biochemical alterations in the hippocampus and amygdala. Footshock-stimulated mice exhibited increased anxiety or a “risk assessment” behaviors, as evidenced by reduced visits to the open arms of the maze or increased duration in the central zone (Walf and Frye, 2007; Szakács et al., 2015), compared to non-footshock 3×Tg-AD mice. Footshock with SRs also induced amyloid deposition, tau hyperphosphorylation, and gliosis in the hippocampus and amygdala of 3×Tg-AD mice. Aβ accumulation in the BLA contributes to increased anxiety in AD transgenic mice (España et al., 2010; Lublin and Gandy, 2010). A strong correlation between microglia activation and tau hyperphosphorylation has also been observed in previous studies (Yoshiyama et al., 2007; Morales et al., 2013). Therefore, behavioral dysfunction may provide insights regarding brain responses to environmental risk factors such as footshock with SRs.

Footshock with SRs exacerbated the progression of the phenotype of 3×Tg-AD mice, as indicated by the impairment of spatial learning and memory. Increases in gliosis, oxidative stress, inflammation, and levels of Aβ, phosphorylated tau protein, and BACE1 and a decrease in the pS9-GSK3β/GSK3β ratio in the hippocampus were also observed in the 3×Tg-AD mice. These results are consistent with recent evidence that chronic mild stress accelerates the onset of AD phenotypes and pathological characteristics (Cuadrado-Tejedor and García-Osta, 2016). Chronic stress results in reactive astrocytes, which promote increased levels of BACE1 (Rossner et al., 2005). Chronic stress, BACE1, and Aβ participate in a positive feedback loop of amyloidogenesis to initiate and sustain a stress response in neurons (O’Connor et al., 2008). Aβ production may be a major connection between oxidative stress and BACE1 (Tamagno et al., 2012). The levels of many antioxidants are negatively regulated by the activity of GSK3β (Farr et al., 2014). Furthermore, oxidative stress increases the levels of phosphorylated tau protein, GSK3β, BACE1, and Aβ in neurodegenerative diseases (Selvatici et al., 2013). Tau is phosphorylated at many sites via several proline-directed protein kinases, including GSK3β and CDK5 (Kimura et al., 2014). However, in the present study, footshock increased the level of GSK3β in 3×Tg-AD mice without interfering with the CDK5 signaling pathway (data not shown). This result is consistent with a previous study that reported that chronic restraint stress elevates GSK3β phosphorylation in mice (Kinoshita et al., 2012). In this study, we also observed that footshock increased iNOS but not COX2, NF-κB, or MAPK (data not shown) in 3×Tg-AD mice. These data are consistent with a previous study that suggested GSK3β activity promotes iNOS induction during chronic inflammation (Cuzzocrea et al., 2006). Evidence also suggests that iNOS enhances glial activation and cytokine release (Diaz et al., 2014). Therefore, we suggest that the deficit in hippocampus-dependent spatial learning and memory mediated by footshock with SRs could be due to an increase in reactive gliosis associated with oxidative stress and inflammation. This deficit may be due to the increase in amyloidogenesis and tau protein phosphorylation resulting from the increase in hippocampal BACE1 levels and GSK3β activation.

Our results also indicate that reduced numbers of cholinergic neurons in the MS/DB and noradrenergic neurons in the LC may significantly contribute to the pathogenesis of the phenotype of footshock-stimulated 3×Tg-AD mice and that NaHS prevents neuronal loss in these regions. A previous study in APP23 mice indicated that norepinephrine depletion reduces the number of cholinergic terminals (Heneka et al., 2006), and a meta-analysis suggested that patients with AD might benefit from a combination of cholinergic and noradrenergic therapy (Lyness et al., 2003). Evidence also suggests that the loss of basal forebrain cholinergic neurons promotes Aβ deposition and tau hyperphosphorylation in the hippocampus in 3×Tg-AD mice (Hartig et al., 2014). The behavioral response of an animal to footshock challenges is dependent on the hippocampus and is mediated by the LC and BLA (Lipski and Grace, 2013). However, neither serotonergic neurons in the raphe nucleus nor pyramidal neurons in the hippocampus differed between mice under footshock or NaHS treatment. These results are consistent with previous studies indicating that the number of serotonergic neurons in the raphe nucleus is not altered by stress (Adamec et al., 2012) and the absence of neuronal loss in the hippocampus in a transgenic model of AD (Cuadrado-Tejedor et al., 2013). Here, we suggest that footshock with SRs reduced the numbers of cholinergic and noradrenergic neurons in association with increased Aβ deposition and tau hyperphosphorylation in the hippocampus; the semi-acute administration of NaHS prevented these footshock-induced effects in 3×Tg-AD mice.

Furthermore, the administration of NaHS attenuated anxiety-like behaviors and the deficit of spatial learning and memory associated with increases in plasma H_2_S, antioxidation, anti-inflammation, BACE1, and pS9-GSK3β as well as with decreases in amyloid deposition, tau hyperphosphorylation, and reactive gliosis in the footshock-stimulated 3×Tg-AD mice. Our finding that NaHS treatment increased plasma H_2_S concentrations and attenuated both emotional dysfunction and cognitive impairment in the footshock-stimulated 3×Tg-AD mice is consistent with the results of previous studies (Chen et al., 2013; He et al., 2014). H_2_S exhibits strong antioxidant capacity and can resist oxidative stress factors such as Aβ_42_ by reducing the BACE1 protein in LPS-induced abnormal pulmonary arteries (Zhang et al., 2011). Hu et al. (2007) reported that the anti-inflammatory effect of H_2_S against LPS-induced activation of microglia and astrocytes was due to inhibition of the iNOS and p38-MAPK signaling pathways in cell culture LPS-induced inflammation. Previous studies have suggested that the administration of NaHS ameliorates Aβ_40_-induced spatial learning and memory impairment, apoptosis, and neuroinflammation at least in part by inhibiting p38 MAPK and p65 NF-κB activity (Xuan et al., 2012; Fan et al., 2013). However, NaHS treatment significantly reduced the level of iNOS but not p38 MAPK or p65 NF-κB in the footshock-stimulated AD mice.

In addition, synthesis of heat shock proteins (HSPs) can be induced by stressful conditions, including heat shock, ischemia, hypoxia, and heavy metals. Especially the substrates of Hsp90 are well-known kinases that are responsible for the phosphorylation of tau, such as GSK3β, CDK5, and Akt. Evidence also shows that Hsp90 induced the production of cytokines, such as IL-6 or TNF-α via both NF-κB and p38 MAPK pathways (Hwang et al., 2004). Evidence further suggests that H_2_S-induced neuroprotection was mediated by Hsp90 against chemical hypoxia-induced injury via anti-oxidant and anti-apoptotic effects (Wuwongse et al., 2010). In our *in vivo* study, the administration of NaHS decreased the level of plasma IL-6 associated with a decrease in iNOS expression, not both NF-κB and p38 MAPK pathways against footshock stimulation. The NaHS treatment also increased the level of activated GSK3β kinase, not CDK5 (data not shown) against footshock stimulation. Therefore, further study of Hsp90 in the NaHS-mediated neuroprotection against footshock stimulation might need a detailed evaluation to better understand the disease pathogenesis and identify therapeutic targets for AD in the future.

Evidence suggests that BACE1 expression was regulated by GSK3β in AD pathogenesis (Ly et al., 2013). Moreover, the amyloid and tau hypotheses are not mutually exclusive; instead, amyloid and tau may concomitantly contribute to neurodegeneration in AD (Ittner and Götz, 2011). In addition, dual-target drugs that modulate both BACE1 and GSK3β might represent a promising therapeutic strategy (Prati et al., 2014). Furthermore, the inhibition of reactive gliosis is a target for therapeutic interventions (Pekny et al., 2014). Therefore, the semi-acute administration of NaHS may act as a neurotherapeutic agent through multiple pathophysiological mechanisms, including antioxidative, anti-inflammatory, anti-reactive gliosis, and reduce amyloid deposition and tau hyperphosphorylation in the multifactorial pathogenesis of AD.

## Conclusion

The present work demonstrated that footshock with SRs induced anxiety-like behaviors and deficits in hippocampus-dependent learning and memory involving pathological changes in the peripheral and central system, including the hippocampus, MS/DB, LC, and BLA regions. Moreover, multiple pathophysiological processes were targeted and attenuated by semi-acute treatment with NaHS in the 3×Tg-AD mice. Therefore, we suggest that exogenous NaHS administration may be a potential therapeutic strategy to alleviate the multipathogenic presentation of AD.

## Author Contributions

The substantial contributions of the authors are described as follows: Conception and design of the study: H-JH and HMH-L. Interpretation of data: H-JH and HMH-L. Acquisition of data: S-LC. Analysis of data: H-JH and HMH-L. Drafting of the article for critically important intellectual content: H-JH and HMH-L. Final approval of the version to be submitted: HMH-L. Agree to be accountable for all aspects of the work and for ensuring that questions related to the accuracy or integrity of any part of the work are appropriately investigated and resolved: H-JH, S-LC, and HMH-L.

## Conflict of Interest Statement

The authors declare that the research was conducted in the absence of any commercial or financial relationships that could be construed as a potential conflict of interest.
